# Feasibility and Preliminary Effects of a Social Media–Based Peer-Group Mobile Messaging Smoking Cessation Intervention Among Chinese Immigrants who Smoke: Pilot Randomized Controlled Trial

**DOI:** 10.2196/59496

**Published:** 2024-07-22

**Authors:** Nan Jiang, Ariel Zhao, Erin S Rogers, Ana Paula Cupertino, Xiaoquan Zhao, Francisco Cartujano-Barrera, Katherine Siu, Scott E Sherman

**Affiliations:** 1 Department of Population Health New York University Grossman School of Medicine New York, NY United States; 2 Department of Public Health Sciences University of Rochester Medical Center Rochester, NY United States; 3 Department of Communication College of Humanities and Social Sciences George Mason University Fairfax, VA United States; 4 Department of Medicine VA New York Harbor Healthcare System New York, NY United States

**Keywords:** smoking cessation, tobacco, mHealth, social media, Chinese American, immigrant, smoking, smoker, mobile messaging, randomized controlled trial, tobacco use, feasibility, acceptability, nicotine replacement therapy

## Abstract

**Background:**

Chinese immigrants experience significant disparities in tobacco use. Culturally adapted tobacco treatments targeting this population are sparse and the use is low. The low use of these treatment programs is attributed to their exclusive focus on individuals who are ready to quit and the wide range of barriers that Chinese immigrants face to access these programs. To support Chinese immigrant smokers at all levels of readiness to quit and address their access barriers, we developed the WeChat Quit Coach, a culturally and linguistically appropriate WeChat (Tencent Holdings Limited)–based peer group mobile messaging smoking cessation intervention.

**Objective:**

This study aims to assess the feasibility, acceptability, and preliminary effects of WeChat Quit Coach.

**Methods:**

We enrolled a total of 60 Chinese immigrant smokers in 2022 in New York City for a pilot randomized controlled trial (RCT) and a single-arm pilot test. The first 40 participants were randomized to either the intervention arm (WeChat Quit Coach) or the control arm (self-help print material) using 1:1 block randomization stratified by sex. WeChat Quit Coach lasted 6 weeks, featuring small peer groups moderated by a coach, daily text messages with text questions, and chat-based instant messaging support from the coach in response to peer questions. The next 20 participants were enrolled in the single-arm pilot test to further assess intervention feasibility and acceptability. All 60 participants were offered a 4-week supply of complimentary nicotine replacement therapy. Surveys were administered at baseline and 6 weeks, with participants in the pilot RCT completing an additional survey at 6 months and biochemical verification of abstinence at both follow-ups.

**Results:**

Of 74 individuals screened, 68 (92%) were eligible and 60 (88%) were enrolled. The majority of participants, with a mean age of 42.5 (SD 13.8) years, were male (49/60, 82%) and not ready to quit, with 70% (42/60) in the precontemplation or contemplation stage at the time of enrollment. The pilot RCT had follow-up rates of 98% (39/40) at 6 weeks and 93% (37/40) at 6 months, while the single-arm test achieved 100% follow-up at 6 weeks. On average, participants responded to daily text questions for 25.1 days over the 42-day intervention period and 23% (9/40) used the chat-based instant messaging support. Most participants were satisfied with WeChat Quit Coach (36/39, 92%) and would recommend it to others (32/39, 82%). At 6 months, self-reported 7-day point prevalence abstinence rates were 25% (5/20) in the intervention arm and 15% (3/20) in the control arm, with biochemically verified abstinence rates of 25% (5/20) and 5% (1/20), respectively.

**Conclusions:**

WeChat Quit Coach was feasible and well-received by Chinese immigrants who smoke and produced promising effects on abstinence. Large trials are warranted to assess its efficacy in promoting abstinence in this underserved population.

**Trial Registration:**

ClinicalTrials.gov NCT05130788; https://clinicaltrials.gov/study/NCT05130788

## Introduction

The prevalence of smoking has declined significantly in the United States over the past 50 years [1]. However, smoking rates remain disproportionately high in socioeconomically disadvantaged populations, including Chinese immigrants (foreign-born Chinese Americans). In New York City (NYC), the city with the largest Chinese immigrant population in the United States, 28% of Chinese American men smoke compared with 18% of the total NYC men [2]. Chinese immigrants, accounting for 68% of the NYC Chinese American population, are more likely to smoke than US-born Chinese Americans (New York City Department of Health and Mental Hygiene, unpublished data, 2018).

Culturally adapted tobacco treatments for Chinese immigrants are sparse [3,4] and the use is low. For example, the National Asian Smokers’ Quitline (ASQ) engages only about 2000 Asian American callers nationwide annually (the number of Chinese-speaking callers is unknown) [5]. The low use of tobacco treatment programs is attributed to 2 major reasons. First, current tobacco treatment programs are designed to offer cessation support to individuals who are ready to quit smoking (ie, they plan to quit within a month). However, only 6%-33% of Chinese Americans who smoke are ready to quit [6-8]. This is largely due to the limited knowledge about the harms of smoking, strong attachment to traditional Chinese prosmoking norms, lack of behavioral capability for quitting, limited social support to facilitate cessation, and low self-efficacy [9-13]. Current tobacco treatment programs often provide no support to move the vast majority of individuals not ready to quit to the stage of being ready to quit. Second, Chinese immigrants have limited access to evidence-based tobacco treatments. Access barriers include limited awareness of treatment resources, skepticism about treatment effects, and time and economic constraints [9-11,14,15]. Less than 20% of Chinese American smokers who attempt to quit use pharmacotherapy or behavioral interventions [7,16]. Research is needed to explore intervention strategies that can engage a broad group of Chinese immigrant smokers, including those not ready to quit, and address the wide range of barriers to cessation.

Over the past decade, social media has been increasingly studied as a tool for tobacco treatment. Social media can reach large populations, allow users to access at their own time of convenience with low or no cost, and may alleviate access barriers to tobacco treatment. A total of 2 systematic reviews have provided evidence supporting the feasibility and acceptability of social media–based smoking cessation interventions [17,18]. In addition, 2 randomized controlled trials (RCTs) conducted among English-speaking smokers compared social media–based tobacco treatment with other cessation support [19,20]. Both interventions feature peer group messaging support, with mixed findings, for example, a Facebook (Meta)–based intervention resulted in abstinence rates comparable to the control at 3, 6, and 12 months [19], whereas a Twitter-based intervention yielded a higher abstinence rate than the control on day 60 [20].

A total of 3 RCTs among Chinese-speaking smokers in Hong Kong [21] and mainland China [22,23] compared WhatsApp (Meta)–based or WeChat (Tencent Holdings Limited)–based interventions with usual cessation care or no tobacco treatment, all using a one-on-one intervention model. A WhatsApp-based intervention led to higher abstinence than the control at 6 months (8% vs 5% biochemically validated 7-day point prevalence abstinence) [21]. A WeChat-based intervention also produced higher abstinence than the control at 26 weeks (12% vs 3% biochemically validated continuous abstinence) [23], but the trial only included individuals ready to quit. Another trial of a WeChat-based intervention did not report any abstinence outcomes [22]. Thus far, the impact of using social media to engage and treat smokers, particularly those not ready to quit, is still unclear.

To reduce tobacco use among Chinese immigrants, our multidisciplinary team developed a culturally and linguistically appropriate social media intervention named WeChat Quit Coach for Chinese immigrants who smoke, across all levels of readiness to quit. Informed by the social cognitive theory and the socioecological model, WeChat Quit Coach addresses multilevel barriers to cessation [24]. The intervention features small, private peer groups moderated by a coach, daily WeChat text messages with text questions, and chat-based instant messaging support from the coach responding to peer questions. By using WeChat, the most widely used social media platform among Chinese with approximately 1.3 billion monthly active users worldwide [25], WeChat Quit Coach holds the potential to reach a large population of Chinese immigrants. In our previous study, we found that 94% of Chinese immigrants who smoke in NYC use WeChat, and that WeChat is used more frequently than other platforms, including Facebook, Twitter, WhatsApp, Line (Line Corporation), and short messaging [10]. This study sought to assess the feasibility, acceptability, and preliminary effects of WeChat Quit Coach among Chinese immigrants who smoke.

## Methods

### Ethics Approval

The study was approved by the Institutional Review Board of New York University Grossman School of Medicine (i20-01959) and preregistered at ClinicalTrials.gov (NCT05130788).

### Study Design

**Figure 1:**
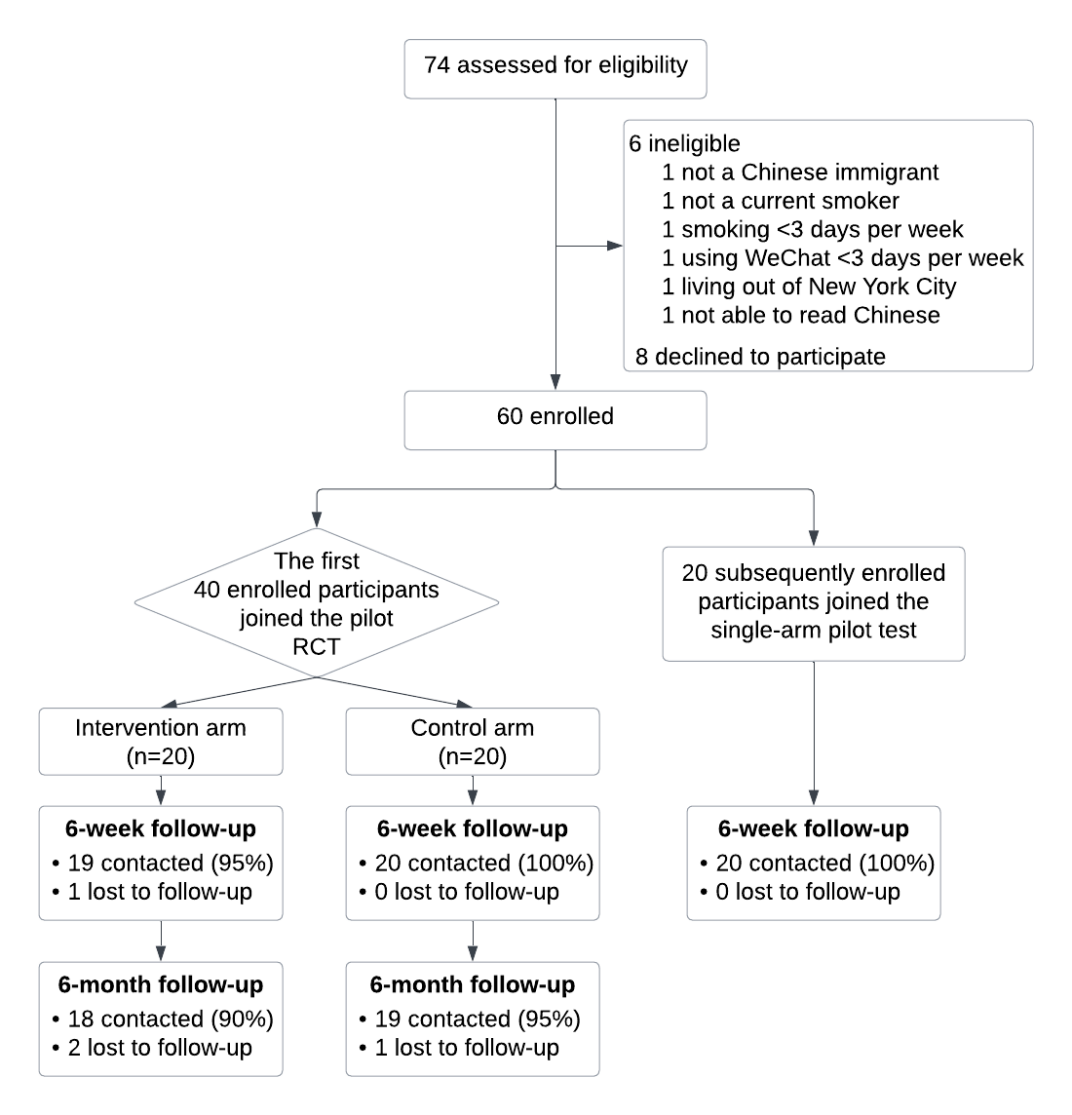
Study schema. RCT: randomized controlled trial.

As shown in Figure 1, we conducted an open-label, 2-arm pilot RCT and a single-arm pilot test among 60 participants. The first 40 participants were enrolled in the pilot RCT. To obtain more data on feasibility and acceptability, we enrolled an additional 20 participants in a single-arm pilot test.

Participants in the pilot RCT were randomized in a 1:1 ratio to the intervention (6-week WeChat Quit Coach) or control arm (self-help print material). Both arms received a 4-week supply of nicotine patches and lozenges by request. Research staff conducted in-person baseline assessment and follow-up phone assessments at 6 weeks and 6 months postintervention initiation and performed biochemical validation through exhaled carbon monoxide (CO) tests at both time points. Participants in the single-arm pilot test received the same treatment as the intervention arm (6-week WeChat Quit Coach and nicotine replacement therapy [NRT]) and completed an in-person baseline survey and a phone survey at 6 weeks. Participants received a US $20 gift card for each survey completed and an additional US $20 for completing each biochemical validation test, irrespective of the test result.

### Participants and Recruitment

Participants were recruited from February to December 2022, primarily in 3 NYC communities that have high concentrations of Chinese immigrants, including Flushing (Queens, NY), Sunset Park (Brooklyn, NY), and Chinatown (Manhattan, NY). Inclusion criteria included (1) self-identified as a Chinese immigrant; (2) age ≥18 years; (3) had smoked at least 100 cigarettes in their lifetime; (4) smoked ≥3 days per week; (5) used WeChat ≥3 days per week; (6) able to read Chinese; (7) lived in NYC; and (8) was somewhat interested in quitting, which was assessed by a question, “Which statement best describes your intention to quit? (A) I don’t want to quit at all; (B) I may quit at some point, but not within the next 6 months; (C) I plan to quit within the next 6 months; (D) I plan to quit within the next 30 days; (E) I am trying to quit.” People who chose an answer other than “A” were considered to be somewhat interested in quitting. Exclusion criteria included (1) current participation in other tobacco treatments, (2) pregnancy or breastfeeding, and (3) inability to connect with research staff through WeChat.

In collaboration with community-based organizations (CBOs), we disseminated study flyers at community events (eg, CBOs’ workshops, health fairs, and immigrant resource fairs) and posted flyers in CBOs’ offices and on their WeChat accounts. The flyer contained information about the study purpose, a study WeChat QR code, and a study phone number. In addition, CBOs and study participants referred potential participants to the research staff.

Potential participants contacted our research staff by scanning the study WeChat QR code or calling the study number. The research staff conducted eligibility screenings over the phone and arranged an in-person study visit for eligible individuals. At the visit, the research staff administered the written consent process and a paper-and-pencil baseline survey. The first 40 participants were enrolled in the pilot RCT. The subsequent 20 participants were enrolled in the single-arm pilot test.

### Randomization

A total of 40 participants were randomized to the intervention or control arm (n=20 per arm) using block randomization stratified by sex (male or female). A randomization module was created and uploaded into REDCap (Research Electronic Data Capture). The research staff performed the allocation. Participants and research staff were unblinded to the assignment.

### Intervention Arm

The research staff created a WeChat peer group every other month. Each group comprised of participants newly enrolled during the 2-month period who were either randomized to the intervention arm or enrolled in the single-arm pilot test (4-10 participants per group), along with a coach (NJ) and a research assistant. During the 6-week intervention period, the coach sent a WeChat text message to the group every day at 9 AM. The messages aimed to (1) enhance motivation to quit by building awareness about the health effects of smoking, quitting methods (eg, quitting preparation and relapse prevention) and cognitive or behavioral tips (eg, coping and refusal strategies), and available Chinese-language tobacco treatment programs (eg, ASQ and local smoking cessation programs); (2) challenge social norms that perpetuate smoking by denormalizing cigarette sharing culture and highlighting the fact that most Chinese immigrants do not smoke; (3) improve self-efficacy through motivational contents and tips for handling slips; and (4) encourage NRT use by addressing misconceptions. Details about the development process of WeChat Quit Coach are available elsewhere [24].

**Figure 2:**
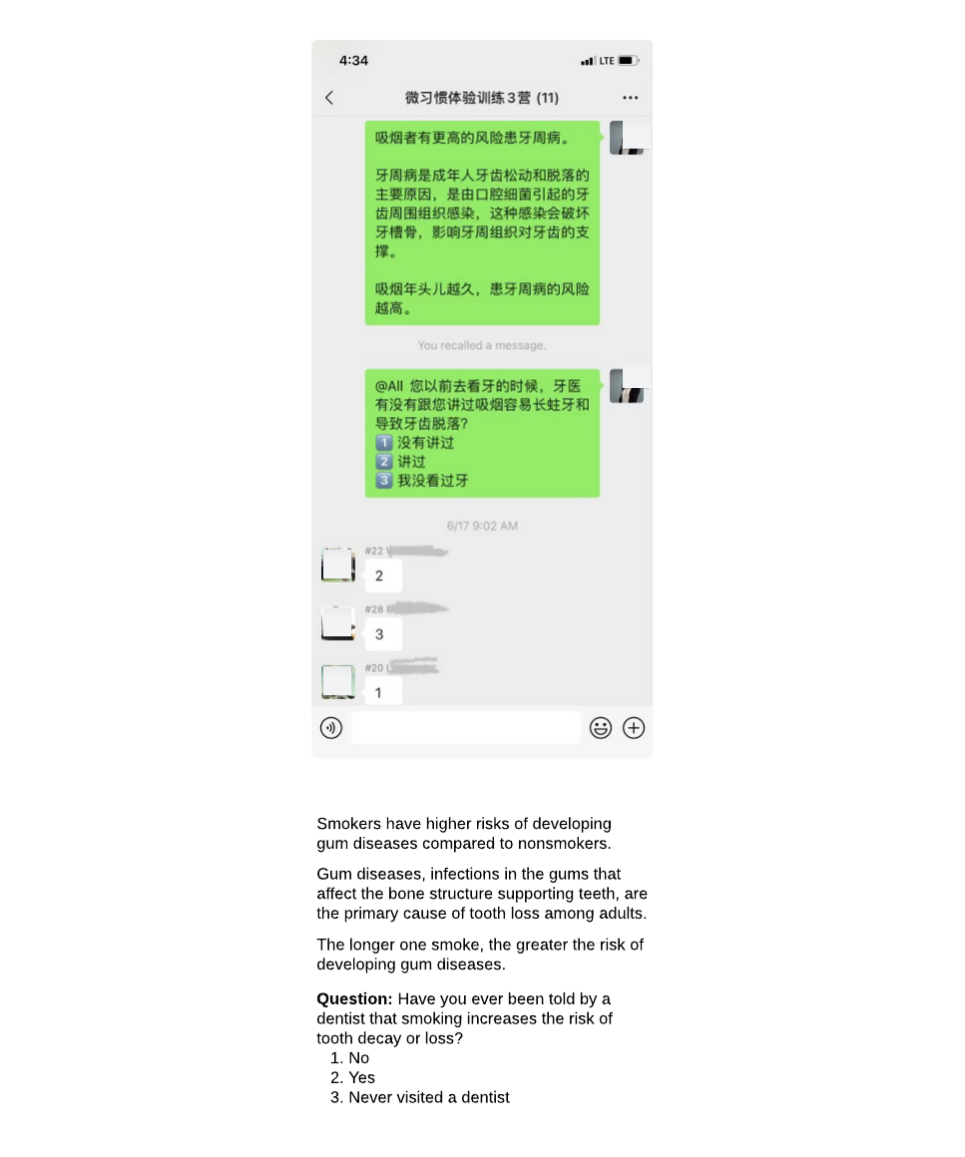
A screenshot of the WeChat Quit Coach intervention.

Following the daily WeChat text message, a text question was sent to the WeChat group each day to promote engagement (eg, “What is the longest period you’ve stayed abstinent in previous quit attempts?”). Participants were encouraged to respond daily and ask their own questions either in the group or directly to the coach, who responded within 24 hours. Figure 2 shows a screenshot of the intervention. If a participant did not respond for 3 consecutive days, a reminder message was sent to him or her through WeChat by the research assistant. Each participant received up to 3 reminders during the intervention. Participants could comment on others’ responses and withdraw from the intervention at any time. The research assistant moderated peer interactions and monitored engagement.

### Control Arm

Participants allocated to the control arm received a leaflet with information about pharmacotherapy and behavioral interventions for smoking cessation, including the ASQ and local Chinese-language tobacco treatment programs.

### NRT Supply

All 60 participants were offered a 4-week supply of free nicotine patches and lozenges. The participants could request NRT medications by texting the study WeChat account or calling the study number, with research staff screening for contraindications over the phone. If no contraindication was reported, participants were recommended a combination of nicotine patches and lozenges with doses as per package instructions. Participants could choose the form (eg, patch, lozenge, or both) and request a lower dose. NRT medications and an instruction sheet (Chinese version) were then delivered by mail or in person.

### Key Measures

Primary outcomes included feasibility, engagement, and acceptability. Feasibility was measured by eligibility rate (defined as the proportion of individuals screened who were eligible), enrollment rate (the proportion of eligible individuals who enrolled), follow-up rate (the proportion of participants who completed follow-up assessment at each time point), and the number of withdrawals. Engagement and acceptability were assessed at 6 weeks among participants from the intervention arm and the single-arm pilot test. Engagement was defined as the number of days participants responded to text questions and the number of participants who posted their own questions for instant messaging support. Acceptability was assessed through Likert scale questions regarding participants’ perceptions about the intervention (eg, the timing of text messages and length of the intervention), helpfulness of intervention components (ie, text messages, text questions, and chat-based instant messaging support), overall satisfaction, and willingness to recommend it to others.

Secondary outcomes, assessed at 6 weeks and 6 months in the pilot RCT, included self-reported and biochemically verified 7-day point prevalence abstinence (yes or no). Participants reporting abstinence in the past 7 days were invited to participate in an exhaled CO test administered by research staff. A CO concentration of ≤6 parts per million indicated biochemically verified abstinence [26]. Additional outcomes encompassed quit attempts (yes or no; defined as no smoking for at least 24 hours because of trying to quit), change in smoking knowledge score, progression to a more advanced stage of change (yes or no), NRT use (yes or no), and use of other tobacco treatment (yes or no). Smoking knowledge was assessed using a 5-item measure adapted from the Global Adult Tobacco Survey [27], “Based on what you know, does smoking cigarettes cause (1) respiratory diseases, (2) lung cancer, (3) coronary heart disease, (4) stroke, (5) diabetes?” (0=“no” or “not sure”; 1=“yes”). The score of smoking knowledge was the sum of the points (ranging between 0 and 5). The stage of change was assessed by a question “Which statement best describes your plan about quitting?” with answer options of 1=“I am not interested in quitting at all” (precontemplation), 2=“I may quit in the future, but not in the next 6 months” (precontemplation), 3=“I plan to quit within the next 6 months” (contemplation), 4=“I plan to quit within the next 30 days” (preparation), and 5=“I am trying to quit now” (action). Participants reporting a more advanced stage of change at follow-up, compared with baseline, were deemed to have transitioned to a more advanced stage.

Other measures included sociodemographic characteristics (eg, age, gender, education, birth country, years of residence in the United States, marital and employment status, and household income level), smoking behaviors (eg, age of smoking initiation, number of smoking days per week, cigarette consumption per day, time to first cigarette in the morning, and current use of other tobacco products), quitting experience (eg, quit attempts in the past 12 months, reasons for past quit attempts, and quitting methods ever used), and smoke-free home rule (“Which statement best describes the rule about smoking inside your home? (1) Smoking is not allowed anywhere in my home [complete rule]; (2) Smoking is allowed in some places or at some times in my home [partial rule]; (3) Smoking is allowed anywhere in my home [no rule]; (4) There are no rules about smoking in my home [no rule]” [28]).

### Statistical Analysis

We performed descriptive statistics to summarize the variables of interest. Secondary outcomes were assessed using an intention-to-treat approach, with missing data treated as no change compared with baseline. To compare the differences between intervention and control arms, effect size estimates were computed, including odds ratios for categorical variables (eg, self-reported and biochemically validated abstinence) and Hedges *g* for the continuous variable (ie, smoking knowledge score) and their 95% CIs. For variables containing zero count cells, odds ratios and 95% CIs were not reported. All data analyses were performed using Stata 17 (StataCorp LLC).

## Results

### Feasibility

Of the 74 potential participants screened, 68 (92%) were eligible, and 60 (88%) enrolled, with no withdrawals. For the pilot RCT, follow-up rates were 98% (39/40 participants) at 6 weeks and 93% (37/40) at 6 months. The single-arm test achieved 100% follow-up at 6 weeks.

### Sample Characteristics

On average, participants were 42.5 (SD 13.8) years old and had resided in the United States for 13.1 (SD 7.7) years (Table 1). Most were male (49/60, 82%), born in mainland China (58/60, 97%), and employed full-time (41/60, 68%). Out of 60, 15 participants (25%) had a middle school education (comparable to US 9th grade) or lower, and 28 participants (47%) reported an annual household income of US $55,000 or less.

**Table 1:** Characteristics of study participants at baseline (N=60).

Characteristics		Pilot RCT^a^		Single-arm pilot test (n=20)	Total (N=60)
		Intervention (n=20)	Control (n=20)		
Age (years), mean (SD)		45.5 (13.5)	42.5 (14.8)	39.6 (13.2)	42.5 (13.8)
Male, n (%)		14 (70)	15 (75)	20 (100)	49 (82)
Years of residence in the United States, mean (SD)		13.5 (7.7)	12.5 (5.1)	13.3 (9.9)	13.1 (7.7)
**Place of birth, n (%)**					
	Mainland China	19 (95)	20 (100)	19 (95)	58 (97)
	Other	1 (5)	0 (0)	1 (5)	2 (3)
**Education, n (%)**					
	Middle school or less	5 (25)	5 (25)	5 (25)	15 (25)
	High school or vocational high school	5 (25)	7 (35)	8 (40)	20 (33)
	Some college, no degree or associate degree	4 (20)	2 (10)	2 (10)	8 (13)
	Bachelor’s or advanced degree	6 (30)	6 (30)	5 (25)	17 (28)
**Marital status, n (%)**					
	Single, never married	5 (25)	2 (10)	9 (45)	16 (27)
	Married, living with a spouse	9 (45)	13 (65)	11 (55)	33 (55)
	Married, living apart with spouse	1 (5)	2 (10)	0 (0)	3 (5)
	Divorced	5 (25)	3 (15)	0 (0)	8 (13)
**Employment status, n (%)**					
	Full-time employed	14 (70)	11 (55)	16 (80)	41 (68)
	Part-time employed	1 (5)	5 (25)	2 (10)	8 (13)
	Other	5 (25)	4 (20)	2 (10)	11 (18)
**Annual household income level, n (%)**					
	Less than US $25,000	6 (30)	5 (25)	2 (10)	13 (22)
	US $25,000-US $55,000	5 (25)	5 (25)	5 (25)	15 (25)
	More than US $55,000	6 (30)	5 (25)	2 (10)	13 (22)
	Not reported or not sure	3 (15)	5 (25)	11 (55)	19 (32)
Age of smoking initiation (years), mean (SD)		19.4 (8.4)	17.2 (4.1)	17 (4.3)	17.9 (5.9)
**Current smoking status, n (%)**					
	Daily smoker	16 (80)	15 (75)	16 (80)	47 (78)
	Nondaily smoker	4 (20)	5 (25)	4 (20)	13 (22)
Cigarette consumption per day, mean (SD)		15.5 (10)	9.4 (6.4)	11.2 (6)	12.0 (8)
**Time to first cigarette in the morning, n (%)**					
	≤5 minutes	9 (45)	2 (10)	3 (15)	14 (23)
	6-30 minutes	2 (10)	6 (30)	6 (30)	14 (23)
	31-60 minutes	2 (10)	1 (5)	4 (20)	7 (12)
	>60 minutes	6 (30)	5 (25)	5 (25)	16 (27)
	No pattern or not sure	1 (5)	6 (30)	2 (20)	9 (15)
Current e-cigarette use, n (%)		5 (25)	8 (40)	10 (50)	23 (38)
**Smoke-free home rule, n (%)**					
	Complete rule	9 (45)	10 (50)	13 (65)	32 (53)
	Partial rule	3 (15)	6 (30)	2 (10)	11 (18)
	No rule	8 (40)	4 (20)	5 (25)	17 (28)
Smoking knowledge score, mean (SD)		2.1 (1.6)	1.9 (1.3)	2.3 (1.8)	2.1 (1.6)
Quit attempts in the past 12 months, n (%)		10 (50)	5 (25)	8 (40)	23 (38)
**Ever use of evidence-based tobacco treatment** ^b^ **, n (%)**		3 (15)	3 (15)	5 (25)	11 (18)
	Used tobacco treatment clinics or programs	0 (0)	0 (0)	1 (5)	1 (2)
	Consulted a family doctor	0 (0)	1 (5)	0 (0)	1 (2)
	Nicotine replacement therapy	3 (15)	3 (15)	4 (20)	10 (17)
	Other smoking cessation medications	0 (0)	0 (0)	0 (0)	0 (0)
	Called a quitline	0 (0)	0 (0)	0 (0)	0 (0)
**Reasons for past quit attempts^b^, n (%)**					
	Concern for my health	14 (70)	13 (65)	13 (65)	40 (67)
	Concern for other’s health	4 (20)	2 (10)	4 (20)	10 (17)
	Family or roommate against smoking	5 (25)	1 (5)	4 (20)	10 (17)
	Smoking is not allowed in my home	1 (5)	0 (0)	1 (5)	2 (3)
	Smoking is not allowed in my workplace	3 (15)	1 (5)	2 (10)	6 (10)
	To set a good example for my child	3 (15)	3 (15)	1 (5)	7 (12)
	To save money	1 (5)	1 (5)	1 (5)	3 (5)
	Other reasons	0 (0)	1 (5)	3 (15)	4 (7)
**Stage of change, n (%)**					
	Precontemplation	11 (55)	9 (45)	4 (20)	24 (40)
	Contemplation	4 (20)	5 (25)	9 (45)	18 (30)
	Preparation	3 (15)	2 (10)	5 (25)	10 (17)
	Action	2 (10)	4 (20)	2 (20)	8 (13)

^a^RCT: randomized controlled trial.

^b^Multiple responses, do not add up to 100%.

In total, 47 out of 60 participants (78%) reported daily smoking. On average, participants started smoking at 17.9 (SD 5.9) years old, smoked 12 (SD 8) cigarettes per day, and had a smoking knowledge score of 2.1 (SD 1.6). Participants were predominantly not ready to quit (42/60, 70% in precontemplation or contemplation stage). While 23 out of 60 participants (38%) reported current e-cigarette use, no participants reported current use of hookah, cigars, or smokeless tobacco (data not shown). Out of 60, 32 participants (53%) reported having a complete smoke-free home rule, 23 participants (38%) reported past 12-month quit attempts, and 11 participants (18%) had used evidence-based tobacco treatment, including 10 participants (17%) used NRT, 1 participant (2%) consulted a doctor, and 1 participant (2%) sought help from a smoking cessation program. None reported ever calling a quitline.

### Engagement and Acceptability

On average, participants responded to WeChat text questions on 25.1 (SD 11) days out of 42 days (median 26.5, IQR 19-33 days). Reportedly, 9 out of 40 participants (23%) sent their own questions to receive chat-based instant messaging support from the coach. In total, 3 out of 40 participants (8%) interacted with peers. One of the participants commented on another’s responses to a text question, while 2 participants each posted a message to communicate with the entire peer group.

Of the 39 participants who completed the 6-week follow-up assessment, 36 participants (92%) were satisfied or very satisfied with the intervention (Table 2). Participants predominantly agreed that WeChat Quit Coach enhanced their motivation to quit (35/39, 90%), increased awareness about how to quit (34/39, 87%), and promoted confidence in quitting (32/39, 82%). Most (32/39, 82%) would recommend the intervention to others, while 8 out of 39 participants (21%) indicated the intervention duration was too short. When asked about perceptions about the intervention, 34 out of 39 participants (87%) agreed that text messages were helpful, 33 participants (85%) indicated that text questions were helpful, and 25 participants (64%) found it enjoyable to answer text questions. Of the 9 who sent their own questions for instant messaging support, 7 participants (78%) reported such support was helpful.

**Table 2:** Acceptability of WeChat Quit Coach among participants (n=39).

Perceptions				Participants, n (%)
**Daily text messages**				
	**Timing of text messages**			
		Too early	5 (13)	
		Too late	0 (0)	
		Just right	21 (54)	
		Does not matter	13 (33)	
	**Length of text messages**			
		Appropriate	29 (74)	
		Too long	5 (13)	
		Other	5 (13)	
	“**Do you find text messages helpful?”**			
		Helpful or very helpful	34 (87)	
		Neither helpful nor unhelpful	4 (10)	
		Unhelpful or very unhelpful	1 (3)	
	“**In general, the messages are easy to understand.”**			
		Agree or strongly agree	36 (92)	
		Neither agree nor disagree	3 (8)	
		Disagree or strongly disagree	0 (0)	
	**Ever showed or sent text messages to others**			10 (26)
**Daily text questions**				
	“**Do you enjoy responding to the text questions?”**			
		Enjoy or very much enjoy	25 (64)	
		Neutral	11 (28)	
		Do not enjoy	3 (8)	
	“**Do you find text questions helpful?”**			
		Helpful or very helpful	33 (85)	
		Neither helpful nor unhelpful	4 (10)	
		Unhelpful or very unhelpful	2 (5)	
**Chat-based instant messaging support^a^**				
	“**Do you find the instant messaging support helpful?”**			
		Helpful or very helpful	7 (78)	
		Neither helpful nor unhelpful	2 (22)	
		Unhelpful or very unhelpful	0 (0)	
**Overall intervention**				
	**Satisfaction with the intervention**			
		Satisfied or very satisfied	36 (92)	
		Neither satisfied nor dissatisfied	3 (78)	
		Dissatisfied or very dissatisfied	0 (0)	
	**Agree or strongly agree with the statements**			
		“It makes me want to quit smoking.”	35 (90)	
		“It is helpful for quitting smoking.”	32 (82)	
		“It makes me feel more confident that I can quit.”	32 (82)	
		“It makes me feel I knew how to quit.”	34 (87)	
		“It makes me want to try again if a quit attempt is unsuccessful.”	30 (77)	
	**Length of the intervention (6 weeks)**			
		Appropriate	30 (77)	
		Too short	8 (21)	
		Too long	1 (2)	
	**Would recommend the intervention to others**			
		Definitely yes	13 (33)	
		Probably yes	19 (49)	
		Probably no	5 (13)	
		Definitely no	2 (5)	

^a^Among participants who sent in their own questions to receive chat-based instant messaging support (n=9).

### Cessation Outcomes

At 6 weeks, 20% (4/20) of intervention participants reported 7-day point prevalence abstinence and were validated biochemically (Table 3). None of the control participants reported abstinence. At 6 months, 25% (5/20) of intervention participants reported abstinence and were validated, while 15% (3/20) of control participants reported abstinence and only 5% (1/20) were validated.

**Table 3:** Intention-to-treat analyses of preliminary effects of WeChat Quit Coach (n=40).

	6 weeks				6 months		
	Intervention arm	Control arm	Effect size estimates^a^, OR^b^ or Hedges *g* (95% CI)	Intervention arm		Control arm	Effect size estimates^a^, OR or Hedges *g* (95% CI)
Self-reported 7-day point prevalence abstinence, n (%)	4 (20)	0 (0)	—^c^	5 (25)		3 (15)	OR 1.89 (0.38-9.27)
Biochemically verified 7-day point prevalence abstinence, n (%)	4 (20)	0 (0)	—	5 (25)		1 (5)	OR 6.33 (0.67-60.16)
Quit attempts, n (%)	11 (55)	7 (35)	OR 2.27 (0.54-9.82)	15 (75)		13 (65)	OR 0.62 (0.16-2.43)
Change in smoking knowledge score, mean (SD)	1.6 (1.6)	−0.3 (1.0)	Hedges *g* 1.10 (0.45-1.79)	—		—	—
Transition to a more advanced stage of change^d^, n (%)	8 (40)	0 (0)	—	7 (35)		5 (25)	OR 1.62 (0.41-6.34)
Use of NRT^e^, n (%)	11 (55)	14 (70)	OR 0.52 (0.12-2.3)	11 (55)		14 (70)	OR 0.52 (0.12-2.3)
Use of other tobacco treatment programs, n (%)	0 (0)	0 (0)	—	0 (0)		0 (0)	—

^a^For categorical variables, we computed the odds ratio and its 95% CI; for the continuous variable, we computed the Hedges *g* and its 95% CI.

^b^OR: odds ratio.

^c^Not applicable.

^d^Compared with baseline.

^e^NRT: nicotine replacement therapy.

Compared with baseline, intervention participants showed an average increase of 1.6 (SD 1.6) points in smoking knowledge at 6 weeks, while the score remained unchanged (mean -0.3, SD 1 points) for control participants. At both follow-up time points, more intervention participants reported quit attempts and progressed to a more advanced stage of change, while more control participants requested NRT during the study period than intervention participants (17/20, 85% vs 16/20, 80%; data not shown in tables) and reported NRT use at both follow-ups (14/20, 70% vs 11/20, 55%).

## Discussion

This is the first study of a social media–based smoking cessation intervention targeting Chinese immigrants. The sociodemographic characteristics of our participants correspond to the overall population traits of Chinese immigrants in NYC, including low income and low education [29]. The study demonstrates the feasibility and acceptability of a WeChat-based smoking cessation intervention and shows promising early efficacy on biochemically verified abstinence at 6 months.

It was found that 70% (42/60) of the participants were in the precontemplation or contemplation stage at enrollment. It suggests that the intervention is attractive to participants, including those not determined to quit in the near term. WeChat Quit Coach targets individuals at all levels of readiness to quit and is implemented through a platform deeply integrated into the daily lives of Chinese immigrants. The intervention has the potential to extend the reach of tobacco treatment within this population.

WeChat Quit Coach created favorable user experiences, as indicated by high levels of acceptability and engagement. Participants were overwhelmingly satisfied with the intervention. The interaction feature (daily text questions) was deemed enjoyable and helpful overall, resulting in a high engagement level compared with other social media peer group cessation interventions [20,30]. It is encouraging that participants post their own questions to receive instant messaging support, resulting in a usage rate (23%) comparable to that of a WhatsApp-based one-on-one cessation intervention for smokers in Hong Kong, which also features instant messaging support (17%) [21]. The results suggest that our intervention generally aligns with the needs of Chinese immigrant smokers. In total, 8 of the 39 participants (21%) reported the intervention was too short, highlighting opportunities for further refinement.

WeChat Quit Coach appears to be promising in promoting abstinence, with a biochemically verified abstinence rate of 25% at 6 months, which is higher than those reported in previous trials of social media interventions for Chinese-speaking smokers in Hong Kong (8% vs 5% biochemically validated 7-day point prevalence abstinence at 6 months [21]) and China (12% vs 3% biochemically validated continuous abstinence at 26 weeks [23]). Other outcomes, including quit attempts and smoking knowledge, were also higher in the intervention arm than control. Larger trials are needed to evaluate the impact of such intervention on abstinence among Chinese immigrants.

A noteworthy finding is that 55% (11/20) of intervention participants and 70% (14/20) of control participants reported NRT use. The high use of NRT could be attributed to the availability of complimentary NRT and the convenient process to request NRT. Chinese immigrants have limited awareness and hold widespread misconceptions about NRT despite its over-the-counter availability [10,14]. Moreover, Chinese immigrants have a high poverty rate compared with immigrants overall in the United States [31]. These factors contribute to the underuse of NRT. Our study indicates that Chinese immigrant smokers are receptive to using NRT when access barriers (eg, cost) are minimized. Our intervention might be more successful in promoting NRT use if cues to try NRT were added to our message library.

This study has several strengths. It is the first to explore a social media–based smoking cessation intervention for Chinese immigrants, a disadvantaged population with high smoking rates. It tests an innovative intervention using a culturally appropriate platform. The study fills gaps in research on tobacco-related disparities and the application of mobile technology for tobacco treatment. It also benefits from the inclusion of both individuals who are ready to quit and those not ready to quit, high follow-up rates, biochemically validated abstinence, and provision of NRT. This study has several limitations. First, as a pilot study aiming to test the feasibility, acceptability, and preliminary effects of our intervention, it was not adequately powered to assess treatment efficacy on abstinence. Second, participants were recruited from NYC, thus limiting the generalizability of our findings to other geographic regions.

This study supports the feasibility of a WeChat-based smoking cessation intervention for recruiting Chinese immigrant smokers across different levels of readiness to quit. The high levels of engagement, acceptability, and promising abstinence outcomes suggest that the intervention may be viable for this population. Larger trials are warranted to determine its efficacy.

## Appendix

Multimedia Appendix 1 CONSORT-EHEALTH (Consolidated Standards of Reporting Trials of Electronic and Mobile Health Applications and Online Telehealth) Checklist.

## Abbreviations

ASQ: Asian Smokers’ Quitline
CBO: community-based organization
CO: carbon monoxide
NRT: nicotine replacement therapy
NYC: New York City
RCT: randomized controlled trial
REDCap: Research Electronic Data Capture

## Data Availability

The data sets generated during and/or analyzed during this study are available from the corresponding author on reasonable request.
